# Cytotoxicity of Titanate-Calcium Complexes to MC3T3 Osteoblast-Like Cells

**DOI:** 10.1155/2016/7895182

**Published:** 2016-12-01

**Authors:** Yen-Wei Chen, Jeanie L. Drury, Joelle Moussi, Kathryn M. L. Taylor-Pashow, David T. Hobbs, John C. Wataha

**Affiliations:** ^1^Department of Restorative Dentistry, University of Washington School of Dentistry, Seattle, WA 98195-7456, USA; ^2^Savannah River National Laboratory, Aiken, SC 29808, USA

## Abstract

Monosodium titanates (MST) are a relatively novel form of particulate titanium dioxide that have been proposed for biological use as metal sorbents or delivery agents, most recently calcium (II). In these roles, the toxicity of the titanate or its metal complex is crucial to its biological utility. The aim of this study was to determine the cytotoxicity of MST and MST-calcium complexes with MC3T3 osteoblast-like cells; MST-Ca(II) complexes could be useful to promote bone formation in various hard tissue applications. MC3T3 cells were exposed to native MST or MST-Ca(II) complexes for 24–72 h. A CellTiter-Blue® assay was employed to assess the metabolic activity of the cells. The results showed that MST and MST-Ca(II) suppressed MC3T3 metabolic activity significantly in a dose-, time-, and cell-density-dependent fashion. MST-Ca(II) suppressed MC3T3 metabolism in a statistically identical manner as native MST at all concentrations. We concluded that MST and MST-Ca(II) are significantly cytotoxic to MC3T3 cells through a mechanism yet unknown; this is a potential problem to the biological utility of these complexes.

## 1. Introduction

Titanium-based materials have been widely explored for use in biological applications. Titanium-oxygen alloys, crystalline titanium dioxide (TiO_2_, anatase), and monosodium titanates (MST) have been the three most widely employed forms of titanium-based materials. Of these, the monosodium titanate particles are the least investigated. Titanium-oxygen alloys have been successfully used for orthopedic hip replacements and dental implants because of their superior physical and biological properties. To enhance bone-bonding bioactivity, titanium alloys have been subjected to alkaline or heat treatments to form a calcium-TiO_2_ layer on their surfaces [1]. The calcium is fixed to the alloy surface and therefore not labile.

Monosodium titanate (MST), one type of titanate, is an inorganic compound of titanium oxide with an amorphous core and crystalline surface that has been modified to create Ti-O-Na groups that have exchangeable sodium [2]. Both MST and crystalline titanium dioxide (anatase) are oxides of titanium but have notable differences in their particle sizes and crystalline surfaces. The well-defined crystalline surface and surface hydroxide groups of the larger (1–20 *μ*m) MST tend to be an ion exchanger rather than smaller (10–25 nm) anatase, which tends to behave as a surface interactor [3]. MST-Ca(II) complex may therefore have utility as a source of Ca(II) in biological systems, sequestered in place by its relatively large particle size.

MST exhibits high affinity for a variety of metal ions at high pH, including strontium ions, which has made it an attractive adsorbent for separating radioactive elements from highly alkaline wastes produced during reprocessing of nuclear fuels [4]. More recently, several groups have explored the possibility of exploiting MST-metal ion affinity in biological applications [2, 4]. Metals such as Au(I), Au(III), and Pt(II) have appeal as candidates for novel drugs because of their unique binding and redox properties [5–10]. However, the systemic toxicity of metals has historically limited their applicability as therapeutic agents [11]. Insoluble monosodium titanate MST particles bound to multivalent metal ions such as Au(III) and Pt(II) could facilitate local delivery by sequestering, via the relatively large particle size of the titanate, metal ions at the site of delivery, thereby reducing systemic toxicity risks. A few studies have reported that titanate-metal compounds amplify antibacterial and anti-inflammatory therapeutic outcomes and reduce the risk of the systemic toxicity of metals [12–15].

Among the titanium-based materials, the biological properties of the titanates are the least studied. The toxicity of native titanates (without metal ions) is important to any use of these materials as therapeutic agents, and the toxicity of MST has been reported [4, 12]. Native MST suppresses mammalian cell metabolism to some extent in several cell types, and some metal ions complexed to MST further suppress cellular metabolism [2, 12, 13, 15]. However, from information available thus far, at lower concentrations, MST toxicity would not preclude its use to locally deliver metal ions in therapeutic applications.

In dentistry, calcium-releasing materials have been used for decades as therapeutic agents to stimulate dentinal repair and bone integration. Calcium hydroxide and mineral trioxide aggregate (MTA) are two such compounds used routinely to encourage reparative dentin formation [16, 17]. Despite proven favorable therapeutic outcomes with these materials, calcium hydroxide and MTA have limitations such as high solubility, weak mechanical properties, high costs, or prolonged setting time [18, 19]. The recent discovery that MST binds and releases Ca(II) makes possible the use of MST to locally deliver calcium ions in dental applications. Titanium alloys have been used for dental endosseous implants because of their superior biocompatibility and mechanical strength [20]. Initially, to enhance the ability of bone to bond to the titanium surface, implants were plasma-sprayed with calcium phosphate [21, 22]. However, debonding of the apatite layer and the inability of these coatings to release calcium from the titanium alloy remain problematic [1]. Subsequently, numerous techniques have been developed to incorporate calcium into titanium surfaces by thermal and chemical treatments, but the calcium in these strategies is not generally releasable [23–25]. Transforming the surface from titanium alloy into an active titanate at least theoretically provides an exchangeable source of calcium on the implant surface. Such a source could be advantageous clinically to osseointegration.

The biological effects on mammalian cells of native titanates and titanates combined with a variety of metal ions (Au(III), Hg(II), Pd(II), Pt(IV), and cisplatin) have been investigated [13]. However, the toxicity of MST-Ca(II) complexes is unknown, particularly to osteoblast-like cells that would be key players in any therapeutic application of calcium-titanate complexes. For this reason, we specifically investigated the cytotoxicity of MST and MST-Ca(II) complexes to osteoblast-like cells* in vitro*. Our goal was to determine the cytotoxicity of MST and MST-calcium complexes using MC3T3 cells, an osteoblast-like cell commonly used to screen for* in vitro* cytotoxicity. Knowing the cytotoxic profile of MST-Ca(II) is a necessary first step in any development of a calcium-releasing dental therapeutic agent.

## 2. Materials and Methods

### 2.1. MST and MST-Ca(II) Loading

MST was obtained from commercial sources (Optima Chemical Group, LLC Douglas, GA). MST was mixed with Ca(II) at a ratio of 6.61 : 1, resulting in a final MST-Ca(II) material with 77 mg of dry Ca(II) per gram of MST. MST-Ca(II) materials were prepared to maximize the loading of calcium onto the MST particulates. MST-Ca(II) was then stored in airtight conical tubes as a 29.67 wt% paste until dilution. For experiments, MST and MST-Ca(II) preparations were mixed with sterile water to obtain stock solutions (4000 mg/L) that were diluted to final concentrations (0–200 mg/L) for experiments.

### 2.2. MC3T3 Cell Culture

MC3T3 osteoblast-like cells (ATCC CRL-2593) were selected because they are a well-characterized osteoblastic cell line with reproducible properties in culture. These cells were cultured in alpha-MEM, supplemented with 10% of FBS, 100 units/mL of penicillin, and 100 *μ*g/mL of streptomycin (all reagents from Life Technologies, Grand Island, NY). Stock cultures of cells were maintained at 37°C, 5% CO_2_, and 100% relative humidity.

MC3T3 cells were plated (5,000 or 30,000 cells/cm^2^) in 96-well format (*n* = 8, flat bottom) in 0.2 mL of culture media per well. The plated cells were incubated for 24 h to allow for adherence before addition of MST or MST-Ca(II) suspensions. MST or MST-Ca(II) complexes were diluted from the stock suspensions and added to each well (0.01 mL into 0.2 mL media) to obtain a final MST concentrations of 0, 0.5, 1, 10, 25, 50, 100, and 200 mg/L. Treated MC3T3 cell cultures were incubated for 24 or 72 h before measuring cellular mitochondrial activity. MC3T3 cells without MST or MST-Ca(II) treatment were used as controls.

### 2.3. Measurement of Cellular Mitochondrial Activity

Cellular mitochondrial activity was estimated using a commercially available CellTiter-Blue® reagent (CTB; Promega, Madison, WI). The CTB assay was selected specifically because our previous work established that, unlike other spectrophotometric assays that rely on optical density (transmittance), the particulate nature of the titanates did not interfere with this fluorescence-based assay [2]. The assay was completed according to the manufacturer's protocol, with an optimized incubation time of 60 min for MC3T3 cells. CTB fluorescence (FL: 560EX/590EM) was measured with a SpectraMax M2 plate reader (Molecular Devices, Sunnyvale, CA). Data were normalized to untreated controls. Statistically significant differences in metabolic activity among controls, MST, and MST-Ca(II) were identified using one-way ANOVA with Tukey* post hoc* analyses (*α* = 0.05).

## 3. Results

### 3.1. Effect of Cell Plating Density and MST Exposure Time

**Figure 1:**
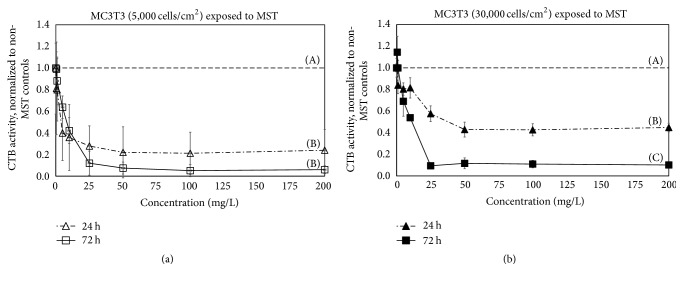
MC3T3 osteoblast metabolic activity (measured by CellTiter-Blue® (CTB)) after* in vitro* exposure to monosodium titanates (MST) at (a) 5,000 cells/cm^2^ or (b) 30,000 cells/cm^2^ for 24 or 72 h. Metabolic activity was normalized to controls without MST (denoted by horizontal dashed line). Lower cell plating densities and increased time of exposure led to depressed cell activity. Uppercase letters (A, B, and C) indicate statistical differences (Tukey pairwise comparisons, *α* = 0.05, and *n* = 8).

MST suppressed MC3T3 cell metabolic activity at concentrations as low as 5 mg/L (Figure 1). Suppression was dose-dependent regardless of the cell plating density and was influenced by the length of MST exposure to the cells. At 5000 cell/cm^2^ (Figure 1(a)), MST-treated cultures reached a plateau of 78% suppression (over untreated controls) after 24 h (50 mg/L). This suppression was statistically significant at doses as low as 5 mg/L (*p* < 0.05, *n* = 8). After 72 h of exposure to MST, MC3T3 cell metabolism dropped even more, 93% relative to controls at 50 mg/L of MST (*p* < 0.05, *n* = 8) and 15% more than suppression at 24 h (Figure 1(a)).

At 30,000 cell/cm^2^ (Figure 1(b)), MST-treated cultures reached a suppression plateau of 57% (versus untreated controls) after 24 h (50 mg/L). After 72 h, the metabolic activity of MC3T3 cells was further and significantly suppressed by MST reaching a plateau of 88% suppression at 50 mg/L (*p* < 0.05, *n* = 8). Thus, the cytotoxic effects of the titanates were less pronounced at the higher plating density.

**Figure 2:**
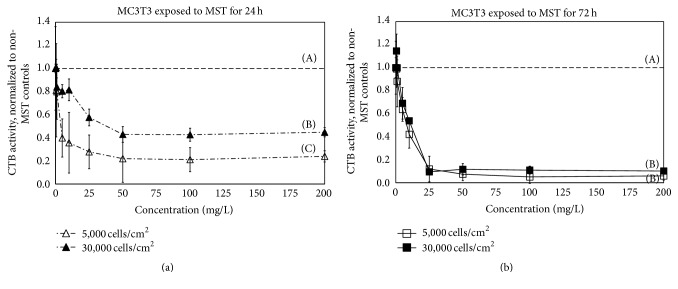
MC3T3 osteoblast metabolic activity (measured by CellTiter-Blue® (CTB)) after* in vitro* exposure to monosodium titanates (MST) for (a) 24 h or (b) 72 h with a 5,000 or 30,000 cells/cm^2^ plating density. Metabolic activity was normalized to controls without MST (denoted by horizontal dashed line). Uppercase letters (A, B, and C) indicate statistical differences (Tukey pairwise comparisons, *α* = 0.05, and *n* = 8).

Time of MST exposure also played a role in how MC3T3 osteoblasts responded to MST (Figures 2(a) and 2(b)). After 24 h (Figure 2(a)) MC3T3 metabolic activity was dependent on cell plating density, but after 72 h (Figure 2(b)) cells were largely independent of initial cell plating density. After 24 h exposure, MST suppressed MC3T3 metabolism 76% at 5000 cell/cm^2^ compared to 56% suppression at 30,000 cell/cm^2^ (Figure 2(a)). This difference was statistically significant (*p* < 0.05, *n* = 8). When the MST exposure period was extended to 72 h (Figure 2(b)), MST suppressed MC3T3 metabolism ~90% regardless of initial cell density.

### 3.2. Effect of MST-Ca(II) Compounds

**Figure 3:**
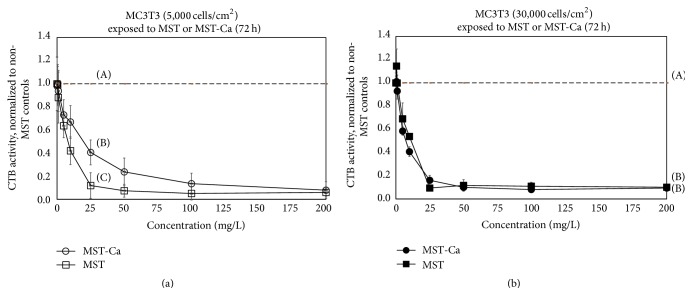
MC3T3 osteoblast metabolic activity (measured by CellTiter-Blue® (CTB)) after* in vitro* exposure to monosodium titanate-calcium compounds (MST-Ca(II)) with (a) 5,000 or (b) 30,000 cells/cm^2^ plating density after 72 h exposures. Metabolic activity was normalized to controls without MST (denoted by horizontal dashed line). Uppercase letters (A, B, and C) indicate statistically different results (Tukey pairwise comparisons, *α* = 0.05, and *n* = 8).

**Figure 4:**
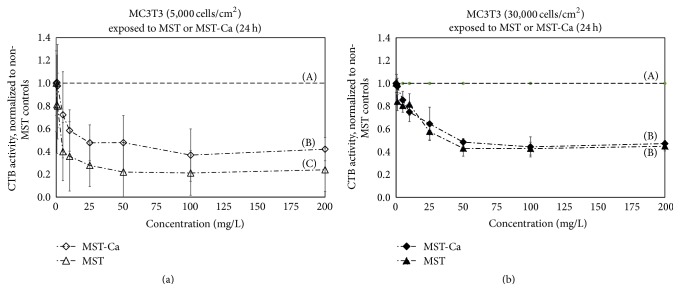
MC3T3 osteoblast metabolic activity (measured by CellTiter-Blue® (CTB)) after* in vitro* exposure to monosodium titanate-calcium compounds (MST-Ca(II)) with (a) 5,000 or (b) 30,000 cells/cm^2^ plating density after 24 h exposures. Metabolic activity was normalized to controls without MST (denoted by horizontal dashed line). Uppercase letters (A and B) indicate statistically different results (Tukey pairwise comparisons, *α* = 0.05, and *n* = 8).

Complexing Ca(II) with MST significantly mitigated native MST suppression of MC3T3 cell metabolic activity at low cell seeding densities (*p* < 0.05, *n* = 8). At 5000 cell/cm^2^ and 72 h incubation (Figure 3(a)), MST-Ca(II) suppressed MC3T3 metabolism nearly 20% less than MST alone. Mitigation of suppression was observed for an intermediate range of titanate concentrations, with no differences between MST and MST-Ca(II) at concentrations below 5 mg/L or greater than 100 mg/L. Results were even more pronounced at 24 h exposure (Figure 4(a)). At 5000 cell/cm^2^ and 24 h incubation, the addition of Ca(II) to MST reduced suppression of MC3T3 cell metabolism by nearly 50% across all concentrations (*p* < 0.05, *n* = 8).

At 30,000 cells/cm^2^, the addition of Ca(II) to the MST did not alter the overall suppression of MC3T3 metabolic activity versus native MST at any concentration (Figure 3(b)). In this case, both MST and MST-Ca(II) suppressed MC3T3 metabolic activity by ~90%. Similar results were observed at 5000 cell/cm^2^ and 24 h incubation (Figure 4(b)), except that suppression was approximately 50% versus untreated controls.

## 4. Discussion

Titanate-metal complexes have been reported to enhance delivery of metal ions to mammalian cells and suppress mammalian cell metabolism to different extents in different cell types [4, 12, 15]. Yet, the cytotoxic profile of native (uncomplexed) titanates is only partly defined. In particular, the potential toxicological effects of titanates and titanate-calcium complexes to MC3T3 cells, a preosteoblastic cell, are underexplored. The osteoblastic cell response is central to the success of many biomedical devices that are implanted into bone or exist juxtaposed to bone or other mineralized tissue such as dentin. Previous studies have reported that calcium ions can be complexed with MST and that calcium ions are released from MST-Ca(II) complexes over time. These reports have led us to propose that MST-Ca(II) complexes may be optimized to enhance Ca(II) release and mineralization of juxtaposed bone or dental pulpal tissues. The first step in testing this hypothesis was to assess the cytotoxicity of MST-Ca(II) complexes, which we have reported in the current study.

Previous studies reported that native MST exhibited relatively low toxicity to mammalian monocytic cells (THP1) and murine L929 fibroblasts [4, 12, 13]. In contrast, others reported that native MST significantly suppressed the mitochondrial activity of rapidly dividing oral squamous-cell carcinoma cells (OSC2) [15]. The OSC2 report hypothesized that native titanates caused greater cytotoxicity to rapidly dividing OSC2 cells than more slowly dividing human THP1 monocytes and murine L929 fibroblasts. Collectively these studies suggested a positive correlation between short population doubling times (PDT) and cytotoxicity [2]. In the current study, native MST suppressed the mitochondrial activity of MC3T3 cells, despite their long PDT (38 h) relative to OSC2 cells (12–15 h) [26]. Suppression was observed in a dose-dependent fashion at both low and high cell seeding densities (Figures 1 and 2). These findings coincide with a previous study which indicated that the cytotoxicity of MST was not restrained to rapidly growing cells but also to slower growing WI-38 fibroblast cells [2]. Based on the current results, PDT does not appear to be an important factor in predicting MST toxicity. It seems reasonable to hypothesize that native titanates have quite different toxicological profiles to different types of mammalian cells. The reasons for these different cellular reactions are not known but may involve the assays used for measuring the cytotoxic effects, the affinity of the titanates for a particular cell type, or the ability of the particles to gain access to the cells once attached.

Initial assays for measuring the effects of MST or MST-metal ion complexes relied on optical density (OD) measurements, particularly the MTT assay [12, 13]. Optical density interference in the MTT assay has been demonstrated due to the particulate nature of titanates, even with attempts to remove particles with centrifugation [2, 3]. The interfering nature of the titanate particulates may underestimate the effects of titanates on cells because the OD results from particulate interference rather than true spectrophotometric absorption. Cell Titer-Blue®, the fluorescence-based assay, was used at this study because previous reports showed that the measurement of mitochondrial function was unaffected by MST particulates. Finally, we note that, in terms of overall assay sensitivity, we have observed no substantial difference between the two methods. Our current results therefore support the utility of this assay for the MST system; the near zero OD of some time points could not have been observed if particulate interference was present (e.g., Figure 2(b)).

Although we cannot provide quantifiable measures, we observed that the MST particles (which refract light substantially in phase contrast view) were always associated with the MC3T3 cells and that washing the cell layer did not remove these particles. Although speculative at this point, the profound suppression of MC3T3 cell metabolism by MST suggests that MST might have better adherence to MC3T3 cells than other cells previously studied. The small size of MST (5–10 *μ*m) particles could make it more accessible to the relatively large MC3T3 cell [4, 27]. The increased contact of the particle-cell interface might intensify the cytotoxic effects of the MST on MC3T3 metabolic activity; this is clearly a direction for further investigation.

The physical disruption of cellular attachment to cell-culture plates could be another mechanism causing high MST cytotoxicity to MC3T3 cells. MST is a heavy particle which settles easily on the bottom of culture wells and has seemingly tenacious adhesion to cell-culture plates. A high density of MST particles occupying sites of adhesion on a cell-culture plate might “compete” for the adherent sites required by the MC3T3 cells for survival or proliferation. In particular, MST-plate attachment might prevent cells from reattaching to the plate postmitosis [2]. Adhesion of particles affecting cell attachment is not without precedent; it has been reported that titanium dioxide particles can disturb fibronectin-mediated adhesion of preosteoblasts [28, 29]. Furthermore, the MST inhibiting effect was more pronounced at the lower initial cell plating density, where the titanates would have had the most access to the plate surface (e.g., Figure 1(a)). Such an effect was not reported with THP1 monocytes, which do not require adhesion for survival and proliferation (anchorage independent) [4, 12]. Future studies should help reach a more complete understanding of the mechanism of titanate cytotoxicity to different mammalian cell types.

In the current study, complexing Ca(II) with MST mitigated MST suppression of MC3T3 mitochondrial activity at low cell seeding densities (Figures 3 and 4). This finding was unexpected and suggested that Ca(II) may interfere with the binding of MST to the MC3T3 cells in some manner. Other studies have reported that MST-Ca(II) complexes release Ca(II) over time. The released calcium ions might interfere with the binding capacity of MST by occupying a limited number of the sites able to attach to cells and trigger toxicity. At high seeding densities, the mitigating effect was not observed and would not have been expected because the effect of the Ca(II) would have been less critical with a more favorable cell-to-titanate ratio. Further experiments will be needed to investigate the affinity of the receptors of the native titanates and the metal-titanates complexes to different mammalian cells.

## 5. Conclusion

The current results show that MST suppressed MC3T3 cell metabolic activity in dose-dependent fashion regardless of the initial cell density. The difference in suppression of MC3T3 cell metabolic activity between different seeding densities was less significant with a longer incubation time. MST-Ca(II) mitigated the suppression of MC3T3 cell metabolic activity at low cell seeding densities and intermediate MST concentrations but not at the high cell seeding densities. In general, MST and MST-Ca(II) are significantly cytotoxic to MC3T3 cells through a mechanism yet unknown.
